# Slower Lower Limb Blood Pooling Increases Orthostatic Tolerance in Women with Vasovagal Syncope

**DOI:** 10.3389/fphys.2016.00232

**Published:** 2016-06-14

**Authors:** Johan Skoog, Helene Zachrisson, Toste Länne, Marcus Lindenberger

**Affiliations:** ^1^Department of Medical and Health Sciences, Linköping UniversityLinköping, Sweden; ^2^Department of Clinical Physiology and Department of Medical and Health Sciences, Linköping UniversityLinköping, Sweden; ^3^Department of Cardiology and Department of Medical and Health Sciences, Linköping UniversityLinköping, Sweden

**Keywords:** syncope, blood pooling, orthostatic intolerance, hemodynamics, baroreceptors, veins, sympathetic nervous system

## Abstract

**Background and Aim:** Slower lower limb blood pooling and associated blunted sympathetic activation has been detected in healthy women prone to orthostatic syncope. Whether these findings are true also for patients with vasovagal syncope (VVS) is unknown. The aim was to investigate initial blood pooling time (pooling_time_, time to 50% of total blood pooling) together with hemodynamic responses and orthostatic tolerance during lower body negative pressure (LBNP) in VVS and healthy controls.

**Methods and Results:** Fourteen VVS women (25.7 ± 1.3 years) and 15 healthy women (22.8 ± 0.8 years) were subjected to single-step and graded LBNP to pre-syncope. Lower limb blood pooling (ml · 100 ml^−1^), pooling_time_ (s), hemodynamic responses and LBNP-tolerance were evaluated. LBNP induced comparable lower limb blood pooling in both groups (controls, 3.1 ± 0.3; VVS, 2.9 ± 0.3 ml · 100 ml^−1^, *P* = 0.70). In controls, shorter pooling_time_ correlated to higher LBNP-tolerance (*r* = –0.550, *P* < 0.05) as well as better maintained stroke volume (*r* = –0.698, *P* < 0.01) and cardiac output (*r* = –0.563, *P* < 0.05). In contrast, shorter pooling_time_ correlated to lower LBNP-tolerance in VVS (*r* = 0.821, *P* < 0.001) and larger decline in stroke volume (*r* = 0.611, *P* < 0.05). Furthermore, in controls, shorter pooling_time_ correlated to baroreflex-mediated hemodynamic changes during LBNP, e.g., increased vasoconstriction (*P* < 0.001). In VVS, pooling_time_ was not correlated with LBNP-induced baroreceptor unloading, but rather highly correlated to resting calf blood flow (*P* < 0.001).

**Conclusions:** Shorter pooling_time_ seems to elicit greater sympathetic activation with a concomitant higher orthostatic tolerance in healthy women. The contrasting findings in VVS indicate a deteriorated vascular sympathetic control suggesting well-defined differences already in the initial responses during orthostatic stress.

## Introduction

Vasovagal syncope (VVS) is defined as a transient loss of consciousness, characterized by a sudden fall in systemic blood pressure (Colman et al., 2004). Women are known to be more affected than men and multiple episodes of VVS could severely compromise quality of life (Colman et al., 2004; Task Force for the Diagnosis Management of Syncope et al., 2009). Although this is a common clinical condition the mechanisms of VVS are not fully elucidated (Mosqueda-Garcia et al., 2000). Upright posture is the major trigger of VVS and gravity-mediated pooling of blood in the peripheral venous capacitance vessels affecting venous return and consequently cardiac output has been proposed to be a major determinant (Liu et al., 2000; Lindenberger et al., 2008; Verheyden et al., 2008; Fu et al., 2012). Conventionally, the main focus has been directed on the amount of blood pooled in the lower limb. However, studies in healthy subjects without a history of VVS have suggested that not the pooled volume, but rather the time by which the hypovolemic stimulus is instituted affects the response to orthostatic stress (Lanne and Lundvall, 1992; Lindenberger and Lanne, 2015). Normally, blood pooling in the lower limbs evokes a compensatory rapid inhibition of cardiac vagal activity followed by baroreflex-mediated sympathetic effects with vasoconstriction in the peripheral vasculature as well as cardiac excitation (Rowell, 1993). Lindenberger and Lanne (2015) found slower lower limb blood pooling in otherwise healthy women experiencing vasovagal reactions during moderate levels of lower body negative pressure (LBNP) compared to hemodynamically stable women. Greater increases in muscle sympathetic nerve activity (MSNA) during a rapid head-up tilt (HUT) have suggested a speed-dependent sympathetic activation (Kamiya et al., 2009). This corresponds to recent findings of enhanced MSNA response during rapid versus slow distension of the occluded venous circulation (Cui et al., 2011). Also during LBNP, rapid initiation of lower limb blood pooling elicited greater cardiovascular compensatory responses, theoretically beneficial both for short and long term cardiovascular regulation during circulatory stress (Lanne and Lundvall, 1992).

Hemodynamic responses to orthostatic stress up to the point of pre-syncope seem to differ between patients with VVS and healthy individuals without a history of syncope (Mosqueda-Garcia et al., 1997). Therefore, it is uncertain if speed-dependent sympathetic activation as described above affects hemodynamic responses equally in patients with VVS. The aim of this study was to investigate the time of initial blood pooling and its effects on orthostatic tolerance and hemodynamic responses during LBNP in women diagnosed with VVS and healthy controls. We hypothesized that women with VVS would present with longer limb blood pooling time, and furthermore that this would be linked with blunted hemodynamic responses to hypovolemic stress as well as to orthostatic intolerance.

## Materials and methods

### Participants

A total of 29 women participated in the study. Some data on these women have been published earlier (Skoog et al., 2015, 2016). Fourteen women (25.7 ± 1.3 years) had previously been screened at Linköping University Hospital for recurrent episodes of unexplained syncope and diagnosed with VVS by means of a typical, positive head-up-tilt (HUT), and otherwise negative screening. HUT was considered positive when syncope was reproduced in association with profound hypotension, bradycardia, or both. Two of the VVS women (14%) had weekly, four (29%) monthly, and eight (57%) yearly problems due to vasovagal reactions. The mean time from onset of first syncope was 7.5 ± 1.0 years. Fifteen healthy women (22.8 ± 0.8 years) without a history of syncope were recruited as control group by means of public advertising at Linköping University. All participants were non-smokers and were not on any regular medication. However, seven of the VVS women used different contraceptives. All participants were studied within the follicular phase of their menstrual cycle or in the low-hormone phase in contraceptive-users (day 1–10). Both VVS and controls were asked to abstain from caffeine and nicotine on the day of laboratory testing and to avoid vigorous-intensity activity the day before the test. All participants were also instructed to ingest 1 L of water the evening before the experiment to ensure proper hydration. The study was approved by the regional ethical review board in Linköping, Sweden, and all subjects signed a written informed consent in accordance with the declaration of Helsinki.

### Lower body negative pressure

All recordings were performed in a temperature-stable room (25°C). Application of LBNP redistributes blood from the upper body to the lower extremities, leading to a central hypovolemia (Cooke et al., 2009). Each subject was in a supine position in the LBNP chamber, tightly sealed at the level of the iliac crest. The negative pressure in the LBNP chamber was generated by a vacuum source, measured by a manometer (DT-XX disposable transducer, Viggo spectramed, Helsingborg, Sweden) and held constant by a rheostat. The LBNP protocol comprised of two experiments. First, after at least 15 min of supine rest the LBNP chamber pressure was reduced by 30 mmHg and maintained for 8 min (LBNP_30_). Second, after at least 15 min of rest an LBNP stress test (LBNP_stress_) was conducted in which the LBNP chamber pressure was reduced by 20 mmHg for 4 min and subsequent reductions in pressure of 10 mmHg were made every 4 min. The test was terminated according to the following criteria: (1) after completion of 4 min LBNP of 70 mmHg; (2) at the onset of presyncopal signs or symptoms (decrease in systolic blood pressure ≥25 mmHg between adjacent 1-min readings, a decrease in diastolic blood pressure of ≥15 mmHg between adjacent 1-min readings, a sudden decrease in heart rate ≥15 bpm, nausea, pallor, profuse sweating, dizziness and/or blurred vision); (3) at the subjects request. LBNP tolerance index (LTI; Lightfoot and Tsintgiras, 1995) was used calculate LBNP tolerance.

#### Calf measurements

**Figure 1:**
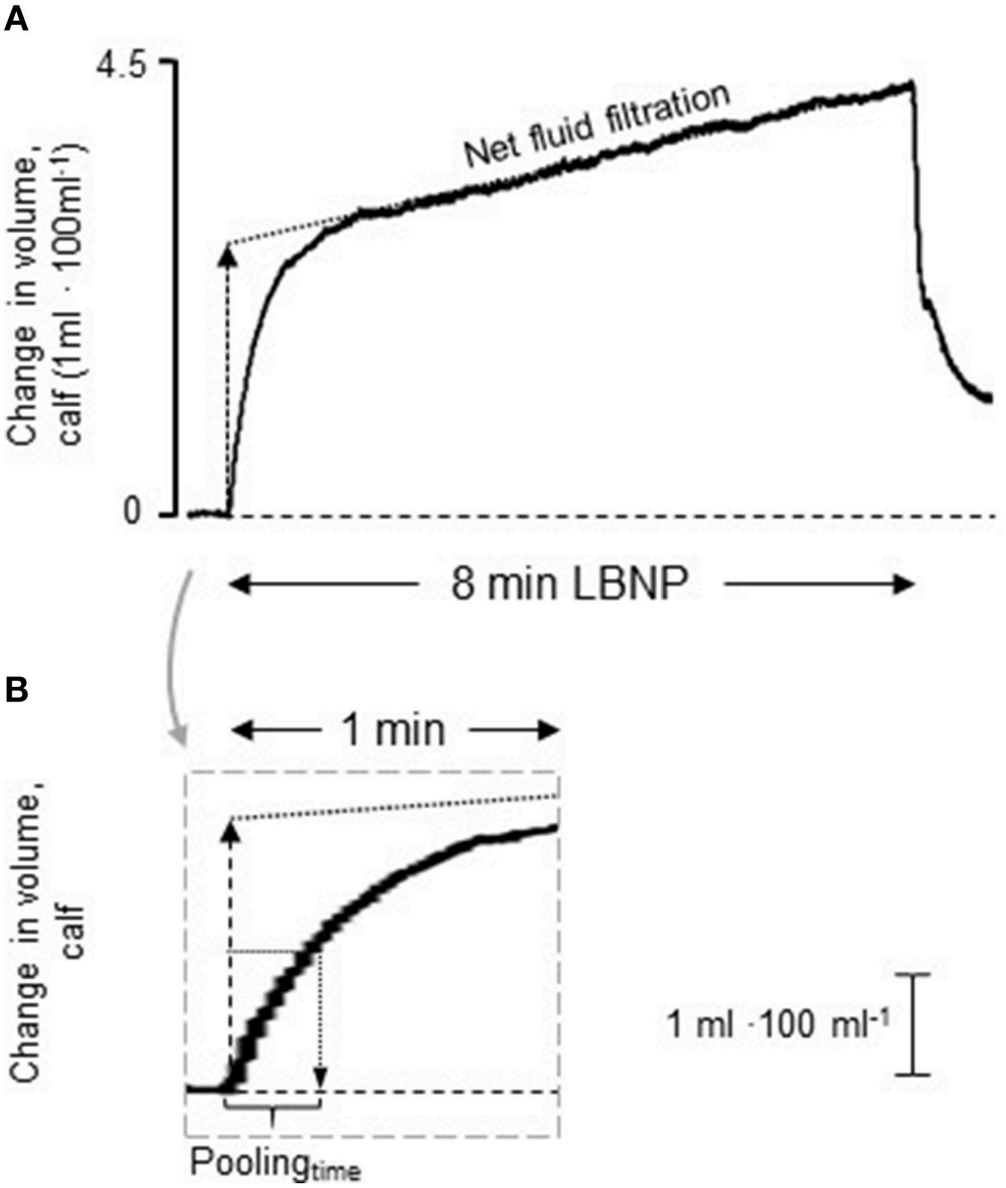
**Representative tracing of volume changes in the calf during lower body negative pressure (LBNP) of 30 mmHg**. **(A)** The rapid initial increase in volume displays blood pooling (calf capacitance response) and the following, slower, increase represents net fluid filtration (dotted line). The capacitance response was calculated by extrapolating the slop of net fluid filtration to the onset of LBNP. **(B)** The gray rectangle shows a magnification of the initial increase in calf volume. The time (s) from LBNP onset to the development of 50% of the capacitance response (pooling_time_) was defined in each individual.

Changes in calf volume (ml · 100ml^−1^) were evaluated with strain gauge plethysmography during LBNP (Hokanson EC-6, D.E. Hokanson, Bellevue, WA). The strain gauge was placed at the maximal circumference of the right calf and the leg was slightly elevated with the heel resting on a foot support with the lowest part of the calf ~2 cm above the floor of the LBNP chamber to avoid external pressure during blood pooling. LBNP-induced calf blood pooling and net fluid filtration were evaluated in accordance with the technique described by Lindenberger and Lanne (2007). In short, LBNP evokes a rapid increase in calf volume, representing venous blood pooling in the calf. This is followed by a slower increase in calf volume, representing net fluid filtration. In order to distinguish between the volume increase attributed to blood pooling and net fluid filtration, calf blood pooling was calculated by extrapolating the slope of net fluid filtration to the onset of LBNP (Figure 1A). The technique has been validated by earlier measurements of volume changes combined with simultaneous recordings of technetium marked erythrocyte activity (Schnizer et al., 1978; Lundvall et al., 1993). Further, the time (s) from LBNP onset to the development of 50% of calf blood pooling (pooling_time_) was defined in each subject (Figure 1B). Pooling_time_ is dependent on the total amount of pooled blood (Lindenberger and Lanne, 2015). However, previous findings indicate similar calf blood pooling in women with VVS and healthy women (Skoog et al., 2016), and total blood pooling during LBNP was evaluated in the present study in order to detect possible group differences.

To evaluate the main determinants of pooling_time_ the following considerations were made; pooling_time_ is dependent on resting arterial blood flow and vascular resistance, where higher blood flow and concurrent lower peripheral resistance are associated with shorter pooling_time_ (Skoog et al., 2015). Further, LBNP-induced blood pooling and baroreceptor unloading leads to sympathetic activation and increased vascular resistance (Bechir et al., 2003; Cooke et al., 2009). Changes in vascular resistance will affect pooling_time_ and it is thus necessary to differentiate between blood flow and vascular resistance at rest and during LBNP. Pooling_time_ may also be related to the maximal amount of blood pooled in the veins as well as venous compliance (Lindenberger and Lanne, 2015). The venous pressure-volume relation appears to shift downwards (lower blood pooling) during sympatho-excitation, possibly by reductions in venous unstressed volume although venous compliance seems unaffected (Greenway et al., 1985; Halliwill et al., 1999). On this basis five parameters were identified and divided in static components (i.e., resting factors); blood flow at rest (I) and venous compliance (II), as well as dynamic components (i.e., factors affected by LBNP-induced baroreceptor unloading); blood flow during LBNP (III), increase in vascular resistance (IV) as well as amount of LBNP-induced blood pooling (V).

All data were recorded, stored and analyzed using the PeriVasc Software (Ekman Biomedical Data AB, Göteborg, Sweden).

### Venous occlusion plethysmography

Venous occlusion plethysmography was used for measurements of calf venous compliance, calf blood flow, and calf vascular resistance at rest. Changes in calf volume was measured in ml · 100ml^−1^. Subjects were placed in the supine position with an acclimatization period of at least 15 min with the right leg slightly elevated and supported at the ankle. A strain gauge was applied at the maximal calf circumference and a cone-shaped, 22-cm-wide thigh cuff was placed on the thigh proximal to the knee on the right leg. The thigh cuff was rapidly (1–2 s) inflated to 60 mmHg using a cuff inflator, occluding venous return from the distal part of the leg (Bergenheim, Elektromedicin, Göteborg, Sweden). After 8 min of venous stasis, cuff pressure was reduced at a rate of 1 mmHg/s.

#### Calf venous compliance

Calf venous compliance (ml · 100ml^−1^ · mmHg^−1^) was calculated from the venous pressure-volume relationship during the deflation phase in accordance to the technique described by Skoog et al. (2016).

#### Calf blood flow and calf vascular resistance

Strain-gauge plethysmography was used to evaluate calf blood flow (ml · 100ml ^−1^ · min^−1^) at rest. Calf blood flow was estimated as the slope of the rapid increase in calf cross-sectional area initiated by thigh cuff inflation over time (Groothuis et al., 2003). Vascular resistance was calculated as mean arterial pressure (MAP) divided by blood flow.

### Cardiovascular measurements

At rest, heart rate and blood pressure were measured with a semiautomatic blood pressure cuff positioned over the brachial artery on the right upper arm (Dinamap Pro 200 Monitor; Criticon, Tampa, FL; USA). Before and during the LBNP protocol, heart rate, and blood pressure were monitored noninvasively, beat-by-beat (Finometer® Midi, Finapres Medical Systems, Amsterdam, the Netherlands). From the continuous blood pressure measurement spontaneous baroreflex sensitivity was determined before the onset of LBNP_stress_ by the validated cross-correlation baroreflex sensitivity (xBRS) method (Westerhof et al., 2004). Aortic outflow was measured from the suprasternal view (Jugulum) using a Vivid E-9 ultrasound scanner (GE Healthcare, Wauwatosa, WI, USA) with a non-imaging 2.5 MHz Doppler probe, presented previously (Waldreus et al., 2013). All measurements were conducted at the same phase in the respiratory cycle (expiration). The recordings were carried out just prior to the start of the LBNP_stress_ and thereafter 2–3 min after each new LBNP level to allow for cardiovascular adjustments to the new LBNP level. Two subsequent aortic outflow measurements, whereby the probe was displaced in between, were conducted at each point of measurement to optimize the detection of the peak velocity integral. The same person (ML) conducted all measurements for all participants. During the aortic flow measurements, the mean valvular velocity time integral (VTI) was registered during three consecutive heart beats, and stroke volume (SV) was calculated as the sub-valvular area × VTI. Cardiac output (CO) was calculated as the product of heart rate (HR) and SV during each measurement. Total peripheral resistance (TPR) was calculated as the ratio between mean arterial pressure (MAP) and CO during each measurement. Lower limb blood flow and maximal LBNP-induced arterial vasoconstriction were estimated during LBNP 30. Since maximal increase in vascular resistance occurs ~30 s after onset of LBNP (Lindenberger et al., 2008), the increase in calf cross-sectional area 25–30 s after LBNP-initiation (strain gauge plethysmography) was used to estimate calf blood flow. Venous filling during the first 30 s (possibly affecting inflow of blood) was similar in VVS and controls (*P* = 0.47), ruling out this confounding factor. Vascular resistance was calculated as mean arterial pressure (MAP) divided by blood flow.

### Cold pressor test

To evaluate the sympathetic reflex arc, the cold pressor test (CPT) was performed in the supine position after at least 10 min of rest. The right foot was immersed into a water bath of 5°C (temperature checked with a digital thermometer just prior to the test) for 120 s. Subjects were instructed to relax, maintain normal breathing, and to avoid isometric muscular contraction throughout the test. Heart rate and blood pressure at rest and during CPT (*at* 40, 80, and 120 s) were measured noninvasively using a semiautomatic blood pressure cuff positioned over the brachial artery on the upper arm (Dinamap Pro 200 Monitor; Criticon, Tampa, FL; US). Forearm blood flow (FBF) was measured by standard venous occlusion plethysmography (Hokanson EC-6, D.E. Hokanson, Bellevue, WA) repeatedly at baseline (x6) and 40, 80, and 120 s (each x2) after the initiation of the CPT, with the arm at heart level and a strain-gauge at the maximal forearm circumference. Blood pressure and forearm blood flow were measured simultaneously, and mean forearm vascular resistance was calculated as mean arterial pressure (MAP) divided by mean FBF at baseline and CPT.

### Statistics

Data are expressed as means ± *SE* unless otherwise indicated. Unpaired Student's *t*-test was used to compare baseline characteristics, lower limb blood pooling and pooling_time_. Linear regression analysis was used to determine any association between pooling_time_ and cardiovascular responses during LBNP_stress_ as well as LBNP tolerance. Multiple linear regressions were used to evaluate the determinants of pooling_time_ in each group. Because of continuous drop-out due to presyncope during LBNP_stress_, between-group differences in cardiovascular responses were tested by unpaired Student's *t*-test with Bonferroni correction for multiple measurements (LBNP 0–40 mmHg). Within-group differences were tested by paired Student's *t*-test with Bonferroni correction for multiple measurements (LBNP 0–40 mmHg). Repeated measure ANOVA with Bonferroni correction for multiple comparisons was used to assess cardiovascular responses during the cold pressor test. *P* < 0.05 were considered statistically significant. Statistical analyses were carried out using SPSS 22.0 for Windows (SPSS Inc., Chicago, Illinois, USA).

## Results

### Subjects characteristics

Table 1 displays baseline characteristics in controls and VVS. Age and anthropometric data were similar between the groups. Further, no differences were found in heart rate, blood pressure, stroke volume, total peripheral resistance, or cardiovagal baroreflex sensitivity. However, calf arterial blood flow was significantly lower in VVS (*P* < 0.01) and calf vascular resistance concomitantly higher (*P* < 0.01).

**Table 1:** **Demographic resting values**.

	**Controls**	**VVS**
*n*	15	14
Age, year	22.8 ± 0.8	25.7 ± 1.3
Height, cm	167 ± 1.4	165 ± 1.9
Weight, kg	64 ± 2.9	62 ± 2.5
BMI, kg/m^2^	22.9 ± 1	22.7 ± 0.8
Calf circumference, cm	36 ± 0.8	36 ± 0.8
Thigh circumference, cm	49 ± 1	49 ± 1.4
HR, beats/min	67 ± 2.6	66 ± 2.7
SBP, mmHg	111 ± 3	104 ± 3.9
DBP, mmHg	65 ± 1.9	63 ± 2.2
MAP, mmHg	82 ± 2.4	76 ± 3.9
CBF, ml/100 ml/min	3.9 ± 0.3	2.6 ± 0.2^**^
CVR, mmHg min/l	22 ± 1.2	34 ± 3.4^**^
SV, ml	76 ± 2.6	68 ± 5.9
TPR, mmHg min/l	21 ± 3.0	25 ± 9.7
BRS, ms/mmHg	20 ± 1.8	19 ± 1.9

*BMI, body mass index; HR, heart rate; SBP, systolic blood pressure; DBP, diastolic blood pressure; MAP, mean arterial pressure; CBF, calf blood flow; CVR, calf vascular resistance; SV, stroke volume; TPR, total peripheral resistance; BRS, baroreflex sensitivity*.

*Mean ± SE*.

** *P < 0.01 controls vs. VVS*.

### Cardiovascular responses to LBNP

**Figure 2:**
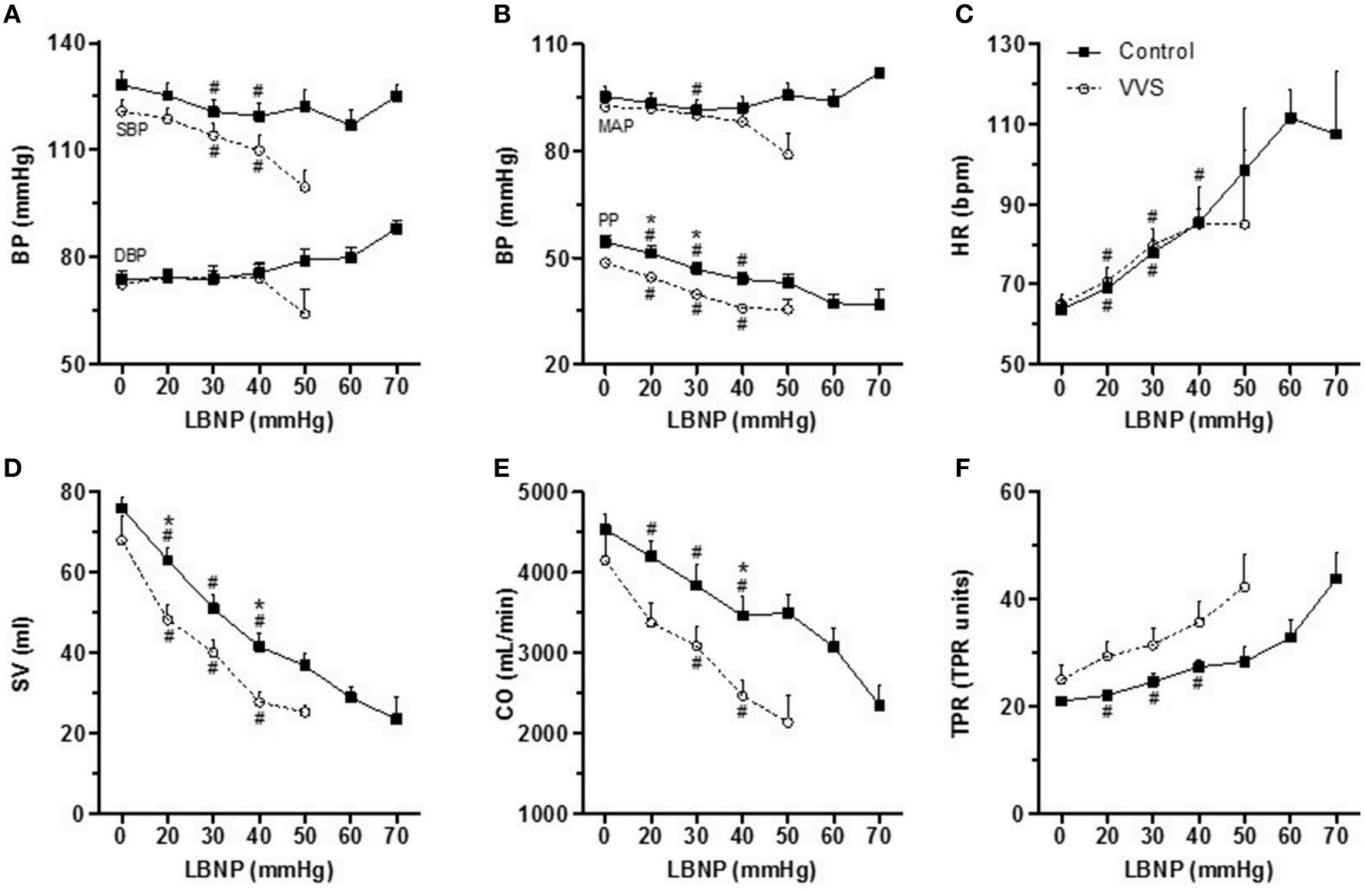
**Cardiovascular responses to LBNP_**stress**_ in controls (black square) and VVS (white circle). (A)** Systolic blood pressure (SBP) and diastolic blood pressure (DBP). **(B)** mean arterial pressure (MAP) and pulse pressure (PP). **(C)** heart rate (HR). **(D)** stroke volume (SV). **(E)** cardiac output (CO). **(F)** total peripheral resistance (TPR). The maximal increase in TPR (difference between resting value and 2 min prior to presyncope) was higher in controls compared to VVS (Controls, 12.8 ± 2.5 mmHg min/l; VVS, 6.4 ± 1.8 mmHg min/l, *P* = 0.05). In controls, the number of subjects are 15 for LBNP 0, 20, 30, and 40 mmHg. Due to presyncope the number decreased to 11 at 50 mmHg, 9 at 60 mmHg, and 2 at 70 mmHg. In VVS, the number of subjects are 13 for LBNP 0, 20, and 30 mmHg. Due to pre-syncope the number decreased to 8 at 40 mmHg and 3 at 50 mmHg. Analyses were performed between LBNP interval 0–40 mmHg. #*P* < 0.05 compared with baseline within the group; *P < 0.05 control vs. VVS.

Figures 2A–F presents cardiovascular responses during LBNP_stress_. Systolic blood pressure (SBP) decreased with increasing LBNP in both controls and VVS (*P* < 0.05). Diastolic blood pressure (DBP) and mean arterial pressure (MAP) displayed an overall stable pattern with no systematic differences between the groups. Pulse pressure (PP) declined in both groups (*P* < 0.05), with VVS presenting with significant lower PP (*P* < 0.05). Heart rate (HR) showed a similar increase in both controls and VVS (*P* < 0.05). Stroke volume (SV) as well as cardiac output (CO) decreased rapidly in both groups (*P* < 0.05), with VVS displaying more pronounced decreases than controls (both *P* < 0.05). Although total peripheral resistance (TPR) seemed to increase in both groups only controls displayed a significant increase in TPR (*P* < 0.05). However, the variability as well as the continuous drop out during lower LBNP levels due to presyncope were greater in VVS (Figure 2F). The maximal increase in TPR during LBNP_stress_ (TPR evaluated 2 min prior to presyncope) was higher in controls compared to VVS (Controls, 12.8 ± 2.5 mmHg min/l; VVS, 6.4 ± 1.8 mmHg min/l, *P* = 0.05).

### Pooling_time_ and LBNP tolerance

The amount of pooled blood volume during LBNP_30_ was 3.1 ± 0.3 in controls and 2.9 ± 0.3 ml · 100 ml^−1^ in VVS, with no differences between the groups (*P* = 0.70). LBNP tolerance index (LTI) was higher in controls (235 ± 8 ΔmmHg · min) than VVS (163 ± 8 ΔmmHg · min, *P* < 0.0001).

**Figure 3:**
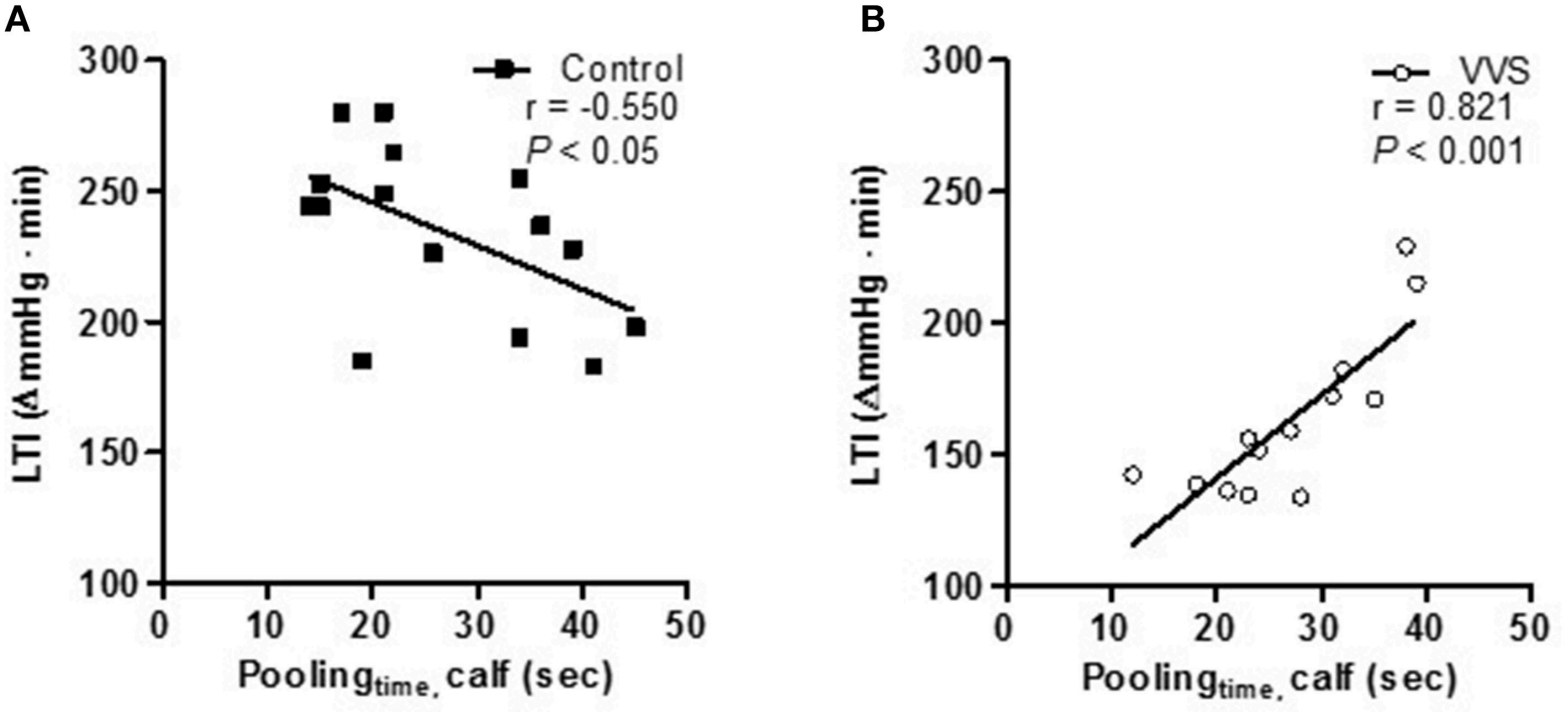
**Correlation between pooling_**time**_ and LBNP tolerance (LTI) in controls (black square) and VVS (white circle). (A)** shorter pooling_time_ correlated to a higher LBNP tolerance in controls (*r* = –0.550, *P* < 0.05). **(B)** shorter pooling_time_ correlated to a lower LBNP tolerance in VVS (*r* = 0.821, *P* < 0.001, control vs. VVS, *P* < 0.001).

Figures 3A,B shows the correlation between pooling_time_ and maximal LBNP tolerance. In controls, shorter pooling_time_ correlated with higher LBNP tolerance (*r* = −0.550, *P* < 0.05, Figure 3A). Conversely, in VVS, shorter pooling_time_ correlated with lower LBNP tolerance (*r* = 0.821, *P* < 0.001, Figure 3B), revealing a reversed relationship between pooling_time_ and maximal LBNP tolerance (control vs. VVS, *P* < 0.001). Despite this, no overall group differences in pooling_time_ were seen during LBNP, being 27 ± 3 and 27 ± 2 s in controls and VVS respectively (*P* = 0.91).

**Figure 4:**
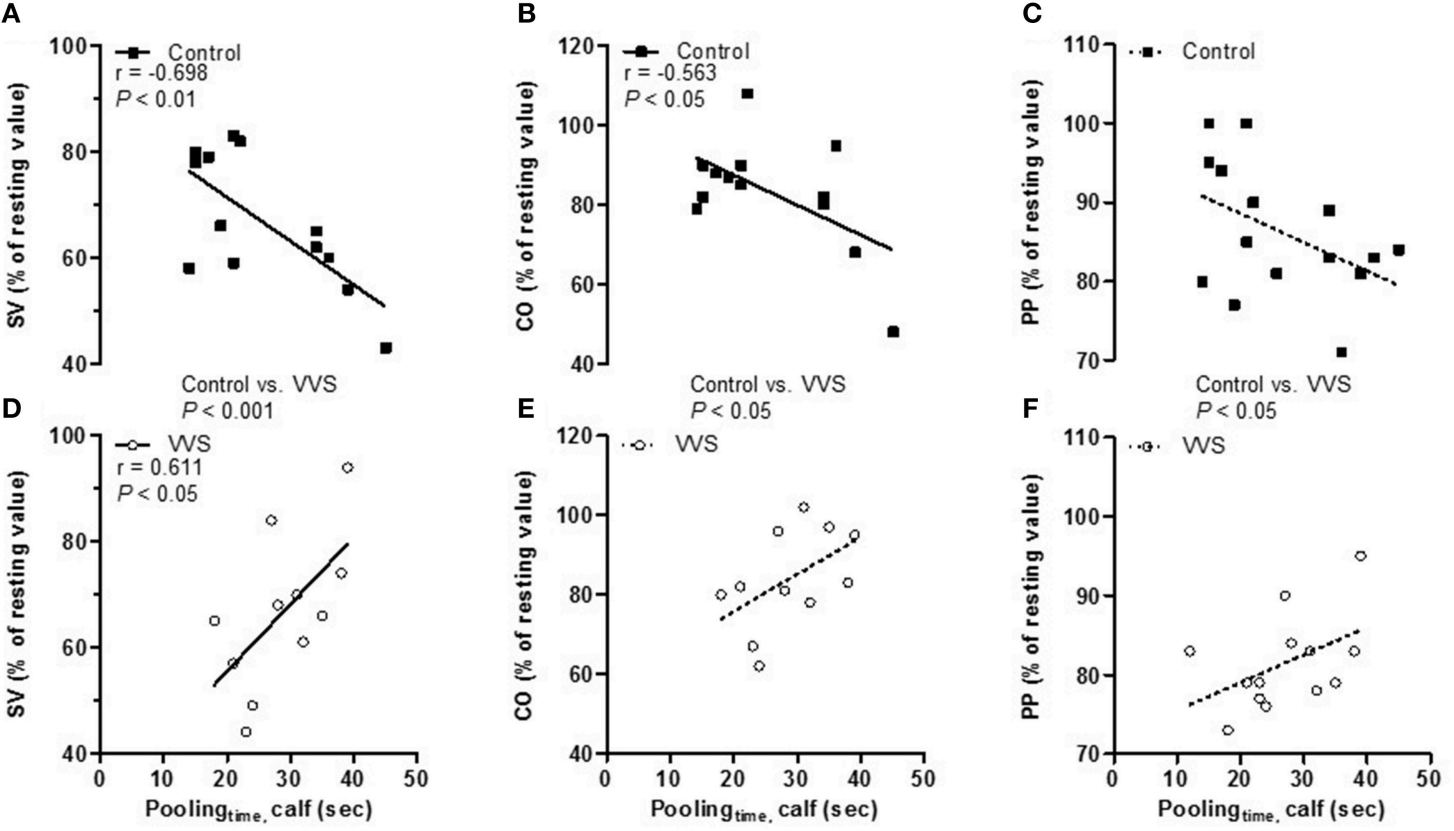
**Correlation between pooling_**time**_ and stroke volume, cardiac output, and pulse pressure (% of resting values) during LBNP_**stress**_ at 30 mmHg in controls (black square) and VVS (white circle). (A)** In controls, shorter pooling_time_ correlated to a better preservation of stroke volume (SV; *r* = –0.698, *P* < 0.01), **(B)** cardiac output (CO; *r* = –0.563, *P* < 0.05), and **(C)** there was a tendency to a better preservation of pulse pressure (PP; *r* = –0.468, *P* = 0.078). **(D)** In VVS, shorter pooling_time_ correlated to greater reductions in stroke volume (SV; *r* = 0.611, *P* < 0.05, control vs. VVS, *P* < 0.001) and **(E)** there was a tendency to greater reductions in both cardiac output (CO; *r* = 0.511, *P* = 0.109, control vs. VVS, *P* < 0.01), and **(F)** pulse pressure (PP; *r* = 0.457, *P* = 0.117, control vs. VVS, *P* < 0.05).

Figures 4A–F depicts the correlation between pooling_time_ and SV, CO as well as PP (% of resting values) evaluated during LBNP_stress_ at 30 mmHg. In controls, shorter pooling_time_ correlated to a better preserved SV (*r* = −0.698, *P* < 0.01, Figure 4A) and CO (*r* = −0.563, *P* < 0.05, Figure 4B). A similar trend was found in PP (*r* = −0.468, *P* = 0.078, Figure 4C). To the contrary, in VVS, shorter pooling_time_ correlated to greater reductions in SV (*r* = 0.611, *P* < 0.05, Figure 4D). Similar trends were also found in CO (*r* = 0.511, *P* = 0.109, Figure 4E) and PP (*r* = 0.457, *P* = 0.117, Figure 4F). The slopes of the regression lines were significantly different between controls and VVS (SV, *P* < 0.001; CO, *P* < 0.05; PP, *P* < 0.05, Figures 4A–F).

### Determinants of pooling_time_

Multiple linear regressions were performed to identify the determinants of pooling_time_ in controls and VVS, see Materials and Methods section (Table 2). We divided the five components into static [i.e., resting factors; calf venous compliance (C_rest_) and calf blood flow (CBF_rest_)] and dynamic [i.e., factors affected by LBNP-induced baroreceptor unloading; calf blood flow (CBF_LBNP_), changes in calf vascular resistance (ΔCVR_LBNP_), and calf blood pooling (CBP_LBNP_)]. The models explained 81% of the variance regarding pooling_time_ in controls (*P* < 0.001) and 86% in VVS (*P* < 0.001). In controls, only dynamic factors, i.e., CVR_LBNP_ (*P* < 0.001), CBF_LBNP_ (*P* < 0.001), and CBP_LBNP_ (*P* < 0.01) contributed significantly to the model. In sharp contrast, only static factors, i.e., CBF_rest_ (*P* < 0.001) and C_rest_ (*P* < 0.05) contributed significantly to the model in VVS.

**Table 2:** **Multiple linear regression for prediction of pooling_**time**_ during LBNP of 30 mmHg**.

**Variables**	**Control**					**VVS**				
	**Adj**. *R*^2^	***B***	***SE B***	**β**	***P***	**Adj**. *R*^2^	***B***	***SE B***	**β**	***P***
CBF_rest_							–7.53	0.92	–0.89	<0.001
C_rest_							1.70	0.64	0.29	<0.05
CBP_LBNP_		7.68	1.95	0.81	<0.01					
CBF_LBNP_		−13.4	2.20	–1.37	<0.001					
Δ%CVR		−0.15	0.03	–0.98	<0.001					
Model	0.809				<0.001	0.859				<0.001

*Adj. R^2^ represents the adjusted proportion of the variance explained by the model. B, unstandardized coefficient; SE B, standard error of the unstandardized coefficient; β, standardized coefficient. CBF_rest_, calf blood flow at rest; C_rest_, calf venous compliance at 30 mmHg; CBP_LBNP,_calf blood pooling during LBNP; CBF_LBNP_, calf blood flow after 30 s of LBNP; Δ%CVR, maximal increase (%) in calf vascular resistance during the initial 30 s of LBNP*.

### Cold pressor test

**Figure 5:**
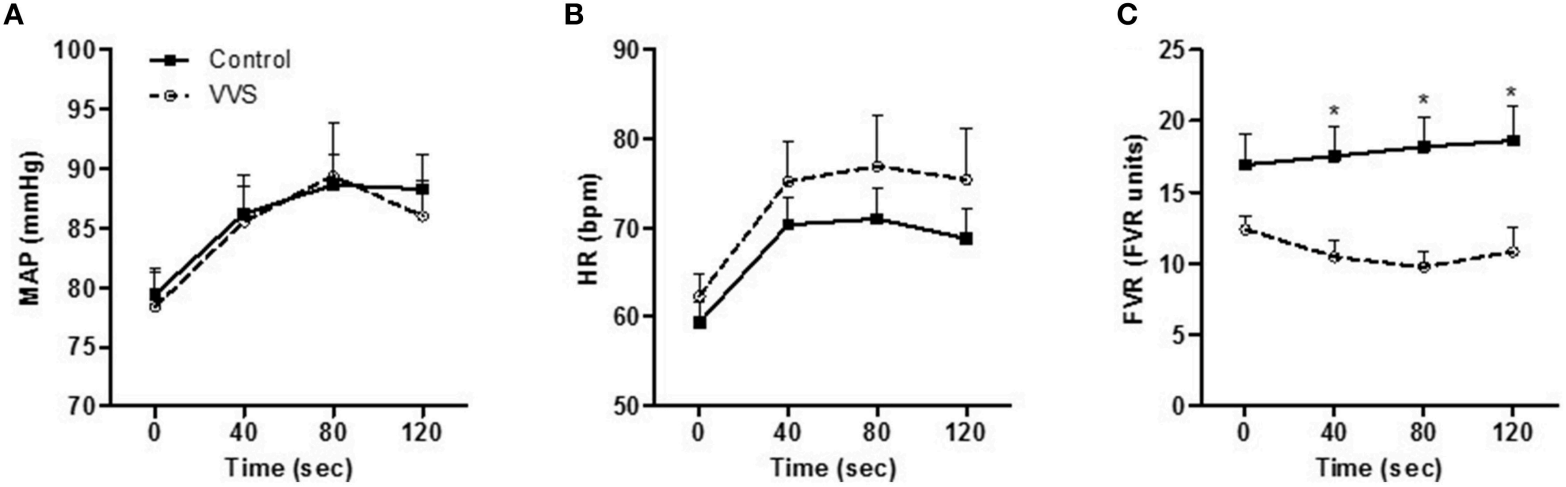
**Hemodynamic responses to cold pressor test in controls (black square) and VVS (white circle). (A)** mean arterial pressure (MAP). **(B)** heart rate (HR) increased in both groups (both *P* < 0.001). **(C)** forearm vascular resistance (FVR) was attenuated in VVS (^*^P < 0.05 control vs. VVS).

Figures 5A–C shows hemodynamic responses during CPT. CPT induced similar increases in MAP (*P* < 0.001, control vs. VVS, *P* = 0.86, Figure 5A) and HR (*P* < 0.001, control vs. VVS, *P* = 0.83, Figure 5B). However, controls responded with greater increase in FVR compared to VVS during CPT (control vs. VVS, *P* < 0.05, Figure 5C).

## Discussion

The main findings in this study were: (1) In controls, shorter pooling_time_, i.e., rapid LBNP-induced lower limb blood pooling correlated with higher LBNP tolerance and with more efficient cardiovascular regulation. In controls, pooling_time_ correlated with baroreceptor-induced changes in blood flow and peripheral vascular resistance; (2) In women with vasovagal syncope (VVS), shorter pooling_time_ correlated with defective cardiovascular compensatory responses and lower LBNP tolerance. Furthermore, in VVS, pooling_time_ was not correlated to baroreceptor-induced responses, but rather resting, static parameters, indicating differences in cardiovascular responses between controls and VVS even during early phases of orthostatic stress.

### Pooling_time_ and orthostatic tolerance

VVS is frequently triggered by orthostatic stress which causes gravitational displacement of blood from the upper body to the venous beds in the lower parts of the body (Groothuis et al., 2007; van Dijk and Wieling, 2013). Conventionally, the main focus has been directed *on the amount* blood displaced into the veins (Grubb, 2005). Although lower body blood pooling is essential for the decrease in venous return during orthostatic stress there is no evidence showing increased blood pooling in the lower limbs of VVS (Stewart et al., 2004; Skoog et al., 2016). Thus, other mechanisms seem responsible for orthostatic intolerance in VVS (Stewart et al., 2004; Lindenberger and Lanne, 2015).

The cardiovascular response to orthostatic stress has been shown to be affected by the speed of the stimulus (Lanne and Lundvall, 1992; Kamiya et al., 2009; Lindenberger and Lanne, 2015). We found pooling_time_ to be similar in controls and VVS. However, the correlation between pooling_time_ and orthostatic tolerance within the groups were totally opposite (Figure 3). The same divergent pattern was also seen within the groups between pooling_time_ and LBNP-induced cardiovascular responses (Figure 4). A recent study found evidence of faster blood pooling to moderate levels of LBNP in a group of hemodynamically stable women compared to women experiencing vasovagal reactions (all women were healthy with no previous history of VVS; Lindenberger and Lanne, 2015). In corroboration, we found that shorter pooling_time_ correlated to higher LBNP-tolerance in healthy controls (Figure 3A). In contrast, shorter pooling_time_ correlated to lower LBNP-tolerance in VVS (Figure 3B). The reliability of this unexpected finding was supported by the associations between pooling_time_ and hemodynamic responses during LBNP_stress_ (Figure 4). More pronounced and/or earlier decreases in SV and CO during LBNP have been linked with orthostatic intolerance in both healthy individuals and patients with VVS (Levine et al., 1991; Jardine et al., 2002; Fu et al., 2004a; Verheyden et al., 2008). In controls, shorter pooling_time_ was associated with better maintained SV and CO (Figures 4A,B). In VVS, shorter pooling_time_ was associated with a greater decline in SV (Figure 4D) and similar trends were found for both CO and PP (Figures 4E,F). A steady decrease in CO, ultimately reaching a critical low limit has recently been suggested as a key factor for VVS (Verheyden et al., 2008; Fu et al., 2012). The more pronounced decrease in PP in VVS may also induce greater cerebral hypoperfusion (Xu et al., 2012).

### Factors correlated with pooling_time_

The components associated with pooling_time_ were significantly different between the two groups (Table 2). In controls, pooling_time_ was associated with dynamic factors, i.e., baroreceptor-induced changes in blood flow, vascular resistance, and blood pooling during LBNP. Shorter pooling_time_ correlated with increased arterial vasoconstriction, indicating speed-dependent sympathetic activation (Table 2). This is in agreement with recent findings from Kamiya et al. (2009) who found greater MSNA responses during rapid compared to slow HUT. The mechanism for the speed-dependent MSNA activation is not clear but animal studies have suggested that the neural arc of the baroreflex has properties of a high-pass filter, i.e., faster changes in blood pressure are transmitted with higher gain (Tank et al., 2007; Kamiya et al., 2009). Further, Cui et al. (2011) identified increased MSNA during venous distension, suggesting possible involvement of afferent type III and IV nerves in the surrounding area of the veins. Rapid pooling_time_ in healthy controls, triggering speed-dependent sympathetic activation, may explain both their efficient cardiovascular regulation during hypovolemia as well as greater LBNP-tolerance (Figures 3A, 4A–C) (Lanne and Lundvall, 1992; Lindenberger and Lanne, 2015).

In VVS on the other hand, pooling_times_ were associated with static factors such as resting blood flow and venous compliance and were unrelated to baroreceptor activity (Table 2). Thus, these novel data point toward attenuated or at least divergent sympathetic responses to LBNP-induced baroreceptor unloading in women with VVS. The present findings of a similar cardiovagal baroreflex sensitivity indicate that VVS have a preserved capacity to modulate cardiac sympathetic tone. However, it should be noted that differences in arterial vasoconstriction control could still be present, as no clear correlation between cardiac and sympathetic baroreflex sensitivity has been shown (Dutoit et al., 2010; Taylor et al., 2015). Attenuated sympathetic baroreflex function has been suggested to account for the incapacity of VVS patients to adequately respond with arterial vasoconstriction during orthostatic stress (Mosqueda-Garcia et al., 1997; Bechir et al., 2003), although the results are not conclusive (Sneddon et al., 1993). To further evaluate the sympathetic reflex arch, responses to cold pressor test (CPT) were measured. The hemodynamic responses to CPT are mediated through efferent sympathetic pathways of the baroreflex loop, while the afferent components are mediated via afferent pain and temperature fibers in the skin connecting to the central nervous system (Victor et al., 1987; Fagius et al., 1989; O'Mahony et al., 2000). Even though CPT induced similar increases in MAP and HR in the two groups, VVS responded with attenuated forearm vasoconstriction (Figure 5), suggesting blunted efferent sympathetic control of the blood vessels (Jardine et al., 2009). This attenuated efferent response of the baroreflex arch in VVS could also implicate why pooling_time_ was associated with resting blood flow and not baroreceptor-mediated hemodynamic responses (Table 2).

### Clinical aspects

Decreased baroreflex-mediated sympathetic control may explain the lower overall LBNP-tolerance in VVS compared to controls, but cannot explain lower LBNP-tolerance within the VVS group in women with shorter pooling_time_ (Figures 3B, 4D–F). Interestingly, in VVS, lower resting blood flow and thus higher vascular resistance were associated with longer pooling_time_ and greater orthostatic tolerance. Although higher vascular resistance at rest may reduce the vasoconstrictor reserve compared to healthy individuals (Fu et al., 2004b) our findings suggest that high vascular resistance improves orthostatic tolerance *within* the VVS group. The mechanisms behind these novel findings are uncertain, but increased sympathetic tone (greater CVR) at rest accompanied with higher arterial vasoconstriction may minimize loss of central blood volume during orthostatic stress in VVS (Figures 3B, 4D–F), particularly when considering their reduced ability to vasoconstrict during sympathetic stimulus (Table 2, Figure 5). Although several pharmacological agents have been studied in VVS the only drug that may be indicated in the ESC guidelines is Midodrine, an alpha-1 adrenergic receptor agonist (Task Force for the Diagnosis Management of Syncope et al., 2009), shown to reduce syncope during HUT (Kaufmann et al., 2002). The rationale for Midodrine is to enhance peripheral vascular tone and the resulting effect mimics the characteristics of VVS women with greater orthostatic tolerance and longer pooling_time_, i.e., Midodrine may bring the patient from the low tolerance end to the high tolerance end as depicted in Figure 3B. Finally, VVS is regarded by many clinicians as a normal physiologic variant in one end of the Gauss curve, i.e., that healthy individuals and frequent fainters respond to orthostatic stimulus in a similar fashion and only differs in the amount of hypovolemic stress required to provoke syncope (Alboni et al., 2007; Jardine, 2013). Our findings challenge this view in numerous ways and a further delineation of the physiology behind these differences, preferably via direct measurements of MSNA and sympathetic baroreflex sensitivity, could provide a new basis for understanding the pathophysiology of VVS.

### Limitations

No healthy women but seven VVS women used contraceptives. However, all measurements were conducted during the first 10 days of the follicular phase, and, as such, during a period with low concentrations of estrogen and progesterone. Furthermore, no significant differences in cardiovascular responses during LBNP were found when normally menstruating women were compare to women using oral contraceptives (Lindenberger et al., 2008), and contraceptives do not seem to have any effect on calf venous compliance and capacitance (Meendering et al., 2005). No second blinded evaluation of the aortic outflow measurements were done. However, all measurements were assessed blinded for both group and LBNP level by a cardiologist experienced in echocardiography (ML). Previous evidence suggest gender differences in both hemodynamic responses and tolerance to LBNP (Convertino, 1998; Fu et al., 2004a) implicate that the present findings cannot directly be transferred to men presenting with VVS.

## Conclusions

In healthy women, rapid LBNP-induced lower limb blood pooling was correlated with higher LBNP tolerance and more efficient cardiovascular responses. Further, pooling_time_ was associated with baroreceptor-induced changes in blood flow and peripheral vascular resistance indicating that speed-dependent baroreflex-mediated sympathetic activation may in part explain individual differences in orthostatic tolerance in healthy women. In women with VVS however, rapid LBNP-induced lower limb blood pooling correlated with lower LBNP tolerance and more deficient cardiovascular responses. Further, pooling_time_ was primarily associated with resting calf blood flow, indicating altered sympathetic vascular control during baroreceptor unloading compared to healthy women. The contrasting findings in pooling_time_ and its association with hemodynamic responses and LBNP tolerance suggest well-defined differences in cardiovascular regulation during orthostatic stress in healthy women and women with VVS.

## Author contributions

JS participated in the study design, performed the experiments, interpreted the data, and drafted the first manuscript. HZ was responsible for study design, interpreted the data and revised the manuscript. TL was responsible for the study design, interpreted the data, and revised the manuscript. ML was responsible for the study design, performed the experiments, interpreted the data, and revised the manuscript.

## Funding

This work was supported by grants from Futurum—the Academy of Health Care, Jönköping County Council; Medical Research Council of Southeast Sweden; the Heart and Lung Foundation, and from the County Council of Östergotland.

### Conflict of interest statement

The authors declare that the research was conducted in the absence of any commercial or financial relationships that could be construed as a potential conflict of interest.

## Abbreviations

C: calf venous compliance
CBF: calf blood flow
CBP: calf blood pooling
CPT: cold pressor test
CVR: calf vascular resistance
FBF: forearm blood flow
FVR: forearm vascular resistance
HUT: head-up tilt
LBNP: lower body negative pressure
LTI: lower body negative pressure tolerance index
MSNA: muscle sympathetic nerve activity
Pooling_time_: time(s) from LBNP onset to the development of 50% of calf blood pooling
VVS: vasovagal syncope.
